# Awareness of Antibiotic Misuse in Upper Respiratory Tract Infections Among Adults in the Bisha Governorate in 2024

**DOI:** 10.7759/cureus.62815

**Published:** 2024-06-21

**Authors:** Mushabab Alghamdi, Atiah Abdullah S Alghamdi, Faisal Ali A Alsalouli, Ali Salem A Alkebiri, Hayf Zayed Z Oraidah, Ahmed Fayi H Alasiri, Ali Hassan Alshamrani, Khalid Mobarak K Alharthi, Ali Wesam A Aldawsari, Ali Mubarak N Almutawa, Ibrahim A Eljack, Mutasim E Ibrahim

**Affiliations:** 1 Internal Medicine, College of Medicine, University of Bisha, Bisha, SAU; 2 Medicine, College of Medicine, University of Bisha, Bisha, SAU; 3 Family and Community Medicine, College of Medicine, University of Bisha, Bisha, SAU; 4 Microbiology, University of Bisha, Bisha, SAU

**Keywords:** antimicrobial resistance, upper respiratory tract infections (urtis), preventive and social medicine, community awareness, overuse and misuse of antibiotics

## Abstract

Introduction: Antibiotics are targeted to kill or inhibit the growth of bacteria and have no effect on viral agents. Unfortunately, viruses cause about 80% of respiratory tract infections, and up to 75% of antibiotics are prescribed for URTIs. Overuse of antibiotics is linked to a number of issues, including the emergence of antibacterial resistance, an increase in the prevalence of chronic illnesses, a rise in the expense of healthcare services, and the emergence of side effects. This study aimed to assess the awareness of antibiotic misuse for URTIs among adults in the Bisha governorate in 2024.

Methods and materials: A community-based, cross-sectional study was conducted in the Bisha governorate among the adult population. Data was collected using an online standardized self-administered adapted questionnaire. The questions vary from multiple choice to Likert scale questions, and each question has 2 points. Data was analyzed using SPSS version 26 (IBM SPSS Statistics, Armonk, NY).

Results: The response rate was about 85.3% (721/845). The ages of the participants ranged between 18 and 75 years. There were 360 (49.9%) male respondents and 361 (50.1%) female respondents. The study revealed that 83.1% (599) of the participants have poor awareness of antibiotic misuse in URTIs. Knowledge of antibiotic misuse consequences was poor at 66.7% (481). There was a significant difference observed between the residents of Bisha city compared to the residents of Bisha villages in total knowledge level about antibiotic misuse in URTIs (p = 0.030).

Conclusion and recommendations: The population of the Bisha governorate has a poor knowledge of antibiotic misuse in URTIs. Therefore, efforts should be made to increase the knowledge and awareness of the general public about the problem.

## Introduction

According to Waksman in 1947, an antibiotic is a chemical compound created by microorganisms that has the power to limit the growth of bacteria and other microorganisms and even destroy them. Today, the term "antibiotic" can mean many things: (I) any antimicrobial substance and (II) any organic molecule of natural or synthetic origin that inhibits or kills harmful bacteria [1]. The development of antibiotics for therapeutic use was undoubtedly the most significant medical advance of the 20th century. Antibiotics not only prevented infectious infections but also made it feasible to treat cancer, do organ transplants, and perform open-heart surgery [2]. In primary care clinics, antibiotics are among the most often prescribed medications for upper respiratory tract infections (URTIs) [3]. Acute infections of the nose, sinuses, throat, and larynx by viruses or bacteria are the cause of upper respiratory tract infections (URTIs) [4]. Since antibiotics only work against bacteria and have no impact on viral agents, they are frequently administered incorrectly to treat viral illnesses including upper respiratory tract infections (URTIs). Generally speaking, URTIs are self-limiting and go away in the same period of time whether or not antibiotics are taken. Therefore, treating these viral illnesses with antibiotics is seen as overusing or misusing them [5]. Adequate administration of antibiotics for URTI is a significant global issue that has not received much attention, both financially and clinically [6]. According to a US survey, 44% of patients with the common cold, 46% with upper respiratory tract infections, and 75% with bronchitis received an antibiotic prescription [7]. It is reported that antibiotics were the drugs most commonly prescribed by primary care physicians for all age groups, representing 40%-63% of the total drug prescriptions in the Asir region in southern Saudi Arabia [8]. The primary reason given by all prescribers and soled for prescribing antibiotics for URTIs was the patient's request for themselves [9]. The impacts include avoidable treatment-related death and morbidity, needless antibiotic side effects, resource waste in the healthcare system, and a rise in bacterial resistance [5].

Problem statement

Clinically and financially, improper antibiotic usage for acute URTI is a significant global issue that has gotten very little attention [6]. Most upper respiratory tract infections (URTIs) are short, mild, and self-limiting, but some can lead to serious complications, resulting in heavy social and economic burden on individuals and society [4]. Although viruses cause about 80% of respiratory tract infections, up to 75% of antibiotics are prescribed for URTI [10]. Adverse effects from antibiotics account for 25% of all adverse drug reactions among hospitalized patients [11]. The overuse of antibiotics is linked to a number of issues, including the emergence of antibacterial resistance, an increase in the prevalence of chronic illnesses, a rise in the expense of healthcare services, and the emergence of side effects [12].

## Materials and methods

Our study is a community-based, cross-sectional study conducted in the Bisha governorate, Asir region. The target population was the adult population who lived in the Bisha governorate, including all males and females, 18 years and older. They must live in the Bisha governorate for at least six months. We excluded all those who refused to participate or who did not meet the inclusion criteria.

Data were collected using an online standardized self-administered questionnaire adapted from Farha et al. [13]. A 2-point Likert scale ranging from "strongly agree" to "strongly disagree" was used to collect data. To overcome the language barrier, a back-translation process was adopted, in which the English version was translated into Arabic by a professional translator before being translated back into English. The same meaning was determined by comparing the original and back-translated versions. The questions vary from multiple choice to Likert scale questions.

The self-administered questionnaire included three sections. The first section included sociodemographic characteristics, age, gender, educational level, and residency. The second section included eight close-ended questions for measuring the knowledge of upper respiratory tract infections and the uses of antibiotics. The third section included four questions measuring the participants' knowledge of the consequences of antibiotic misuse.

The sampling technique was non-probability, convenience sampling.

Data were collected using Google Forms (Google, Inc., Mountain View, CA) and exported to Excel version 2010 (Microsoft Corp., Redmond, WA). Data were then exported, encoded, and analyzed using SPSS version 26 (IBM SPSS Statistics, Armonk, NY). Basic variables were expressed as the mean ± standard deviation (SD) and frequency tables. Data were presented in percentages (%) and depicted with charts, graphs, and tables. A chi-square test was done and used to analyze categorical data. The results are considered statistically significant when the p value is <0.05.

Ethical approval was obtained from the University of Bisha Institutional Review Board (IRB) before data collection. Before completing the surveys, participants were made aware of the goals of the study, and their consent was sought. Additionally, the participants were made aware of their freedom to leave the study at any moment. The study maintained confidentiality at all times, guaranteeing the security and privacy of the respondents' information.

## Results

A total of 845 participants completed the questionnaire. We were left with 721 participants after the exclusion of 124 participants as they did not meet the inclusion criteria. The participants' ages ranged between 18 and 75 years. The mean age of the participants was 33.88 ± 12.7. Sex distribution was almost equal, with 360 males (49.9%) and 361 females (50.1%). Most of the respondents' level of education was bachelor/diploma at 74.9% (n = 540), followed by high school at 18.7% (n = 135), and postgraduate at 6.4% (n = 46). The majority of the respondents were residents of Bisha (n = 561, 77.8%), and 160 (22.2%) lived in Bisha villages (Table 1).

**Table 1:** Sociodemographic characteristics of the study population This table represents the data in N with its percentage (%) and mean ± SD. N: number, SD: standard deviation

Parameter	Variables	Frequency
Age	Mean ± SD	33.88 ± 12.7
Gender	Male	360 (49.9%)
	Female	361 (50.1%)
Education level	High school	135 (18.7%)
	Bachelor/diploma	540 (74.9%)
	Postgraduate	46 (6.4%)
Residence	Bisha city	561 (77.8%)
	Bisha villages	160 (22.2%)

Regarding the knowledge about URTIs, only 207 (28.7%) correctly identified tonsillitis as a URTI. One hundred twenty-eight (17.75%) have chosen all answers to the question about typical symptoms of URTI. The majority of the respondents (n=508, 70.5%) had good knowledge when asked about the main cause of URTI by choosing viruses, 204 (28.3%) stated that bacteria is the main cause, and fungi and parasites had the least frequency of 5 and 4, respectively (Table 2).

**Table 2:** Knowledge of URTIs This table represents the data in N with its percentage (%). URTIs: upper respiratory tract infections, N: number

Question	Variables	N (%)
Which of the following is a URTI?	Pneumonia	212 (29.4%)
	Bronchitis	223 (30.9%)
	Asthma	79 (11%)
	Tonsillitis	207 (28.7%)
What are the most typical symptoms of URTI? (Multiple choices allowed)	Runny nose	344 (47.7%)
	Sore throat	425 (58.9%)
	Cough	536 (74.3%)
	Fever	311 (43.1%)
What do you think the main cause of URTIs is?	Bacteria	204 (28.3%)
	Viruses	508 (70.5%)
	Fungi	5 (0.7%)
	Parasite	4 (0.6%)

The level of knowledge of the definition and uses of antibiotics was evaluated using a Likert scale (Table 3).

**Table 3:** Knowledge of the definition and uses of antibiotics This table represents the data in N with its percentage (%). SD: strongly disagree, D: disagree, N: neutral, A: agree, SA: strongly agree, N: number

Questions/scale	SD (N (%))	D (N (%))	N (N (%))	A (N (%))	SA (N (%))
Antibiotic is a medicine that can kill or inhibit the growth of bacteria.	11 (1.5%)	30 (4.2%)	98 (13.6%)	327 (45.4%)	255 (35.4%)
Most URTIs do not need antibiotics.	21 (2.9%)	175 (24.3%)	189 (26.2%)	227 (31.5%)	109 (15.1%)
People who suffer from flu-like symptoms recover more quickly if treated with antibiotics.	28 (3.9%)	106 (14.7%)	125 (17.3%)	302 (41.9%)	160 (22.2%)
Antibiotic use can prevent complications from URTIs.	16 (2.2%)	67 (9.3%)	137 (19%)	336 (46.6%)	165 (22.9%)
When I have a fever, I expect the doctor to prescribe me an antibiotic.	42 (5.3%)	172 (23.9%)	110 (15.3%)	250 (34.7%)	144 (20%)

**Figure 1:**
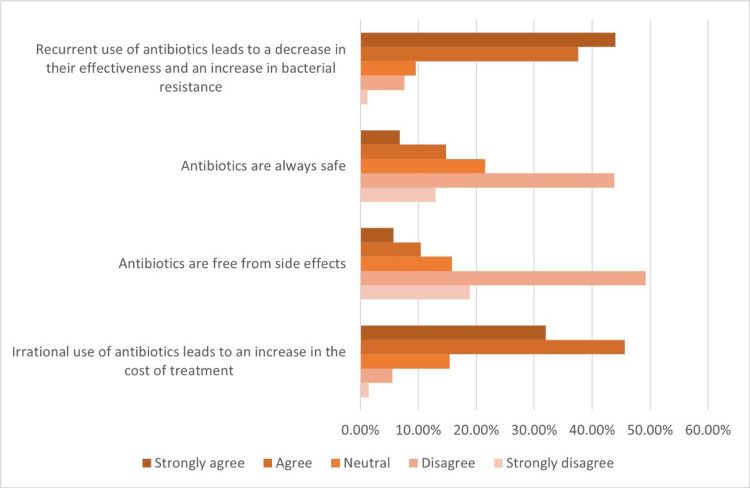
Knowledge of antibiotic misuse consequences This figure represents the numbers, and each color indicates an answer depending on the explanation below the figure.

Also, the level of knowledge of the consequences of antibiotic misuse was evaluated using a Likert scale, and the results are shown in Table 4 and Figure 1.

**Table 4:** Knowledge of consequences of antibiotic misuse This table represents the data in N with its percentage (%). SD: strongly disagree, D: disagree, N: neutral, A: agree, SA: strongly agree, N: number

Questions/scale	SD (N (%))	D (N (%))	N (N (%))	A (N (%))	SA (N (%))
Recurrent use of antibiotics leads to a decrease in their effectiveness and an increase in bacterial resistance.	9 (1.2%)	55 (7.6%)	69 (9.6%)	271 (37.6%)	317 (44%)
Antibiotics are always safe.	94 (13%)	316 (43.8%)	155 (21.5%)	107 (14.8%)	49 (6.8%)
Antibiotics are free from side effects.	136 (18.9%)	355 (49.2%)	114 (15.8%)	75 (10.4%)	41 (5.7%)
Irrational use of antibiotics leads to an increase in the cost of treatment.	10 (1.4%)	40 (5.5%)	111 (15.4%)	329 (45.6%)	231 (32%)

**Figure 2:**
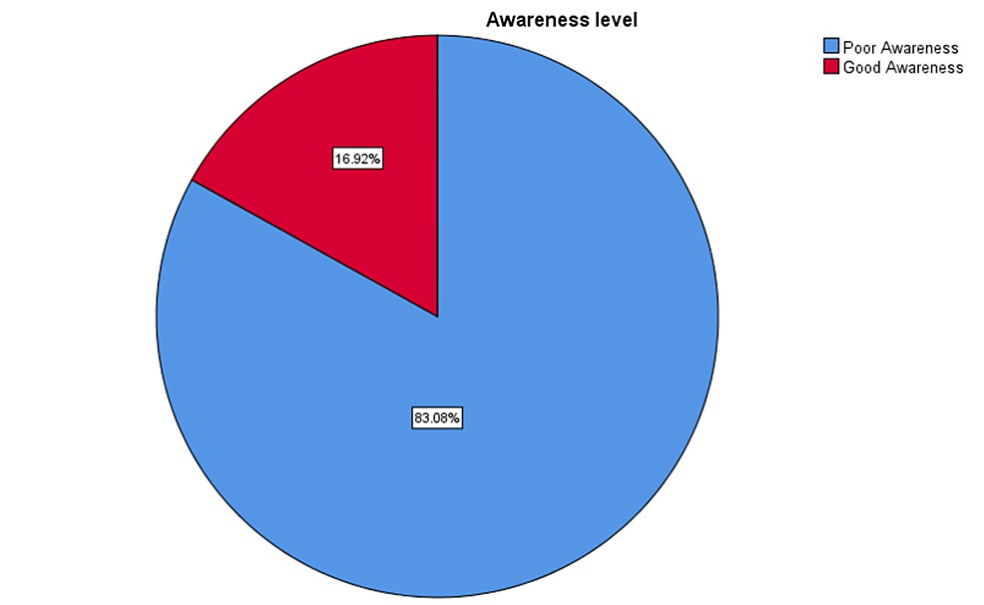
Total level of awareness of antibiotic misuse in upper respiratory tract infections among adults The blue color indicates good awareness. The red color indicates poor awareness.

The data of this study about the total knowledge of antibiotic misuse in upper respiratory tract infections (URTI recognition, antibiotic indications, and consequences) was analyzed. The result revealed that 599 (83.1%) respondents have poor knowledge, with a score of less than 2.6 out of 5. On the other hand, only 122 (16.9%) respondents have good knowledge, with a score equal to or more than 2.6 out of 5 (Figure 2).

**Figure 3:**
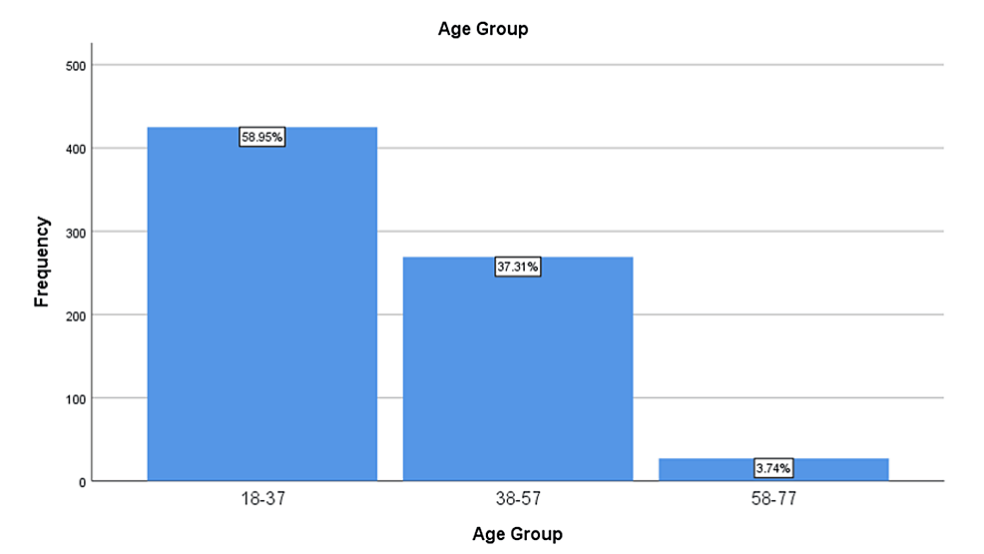
Distribution of age groups This figure represents the number of participants in each age group.

Also, we classified the participants into three age groups with 20 years interval to ease the analysis process (Figure 3).

A chi-square test was done using the database software program SPSS, and we found no association between sociodemographic factors and the level of awareness, except with the residence (Table 5).

**Table 5:** Total knowledge assessment on antibiotic misuse in upper respiratory tract infections among adults This table represents the data in N with its percentage (%), chi-square value, and p value. *Significant (p ≤ 0.05) **Non-significant (p > 0.05) N: number

Sociodemographic variables	Poor knowledge (N (%))	Good knowledge (N (%))	Chi-square value	p value
Age group				
18-37	349 (82.12%)	76 (17.88%)	2.079	0.354**
38-57	225 (83.63%)	44 (16.37)		
58-77	25 (92.59%)	2 (7.41%)		
Gender				
Male	290 (80.56%)	70 (19.44%)	3.257	0.071**
Female	309 (85.60%)	52 (14.40%)		
Educational level				
High school	112 (82.96%)	23 (17.04%)	3.002	0.223**
Bachelor/diploma	453 (83.89%)	87 (16.10%)		
Postgraduate	34 (73.91%)	12 (26.09%)		
Residence				
Bisha	457 (81.46%)	104 (18.54%)	4.704	0.030*
Bisha villages	142 (88.75%)	18 (11.25%)		

## Discussion

In our study, we assessed the awareness of the adult population in Bisha governorate about antibiotic misuse in upper respiratory tract infections. The overall responses were 845 participants. A total of 721 (85.3%) participants were included in the study, and 124 of them were excluded according to our criteria. We found that 83.1% (n = 599) of the participants revealed having poor knowledge. With this in mind, we evaluated factors that may affect the population's awareness so that these factors can be targeted in future population-specific awareness campaigns. This assessment may help effectively utilize the tool, ultimately facilitating the accomplishment of the goals set by the WHO and MOH to prevent and combat antibiotic resistance.

Our findings could be explained by the fact that the participants' ages did not affect their level of awareness. Indeed, although no previous studies reported their results in the same way as done here, there are similarities between our findings and what is reported in the literature. For example, Alqarni et al. [14] reported that knowledge scores were lower in the 18- to 30-year-old range, a finding that was related to older participants having a higher level of education. Interestingly, however, this was not the case for the results reported here, with education and age showing no apparent link to the level of awareness.

Regarding the typical symptoms of upper respiratory tract infections, only 17.1% (n = 123) have full awareness and 40.3% (n = 290) expect more than half. The final result of these questions indicates poor knowledge, and it was the same as the study conducted by Nguyen et al. [15] in India.

Regarding the main cause of URTIs, the feedback from our participants showed good knowledge. This was similar to the study conducted by Elberry [16] in Jeddah, Saudi Arabia, in 2011, which showed that 71.85% had good knowledge from a total of 199 participants.

Of the respondents, 46.60% (n = 336) strongly agreed or agreed with the statement "most URTIs do not need antibiotics." The result is quite consistent with the findings of a study aimed to evaluate parents' awareness toward antibiotic use in upper respiratory tract infection in children in the Al-Qassim region of Saudi Arabia, in which similar statements were used in the evaluation such as "most respiratory infections will relieve without using any antibiotics," in which more than half of the parents (52.3%) strongly agreed or agreed. Of the parents, 18.5% strongly agreed and 26.4% agreed (44.9% in total) with the statement "most of the URTIs are caused by viruses, and therefore, antibiotics should not be used" [17].

Another study conducted by Alrafiaah et al. [18] in Saudi Arabia assessing the knowledge of parents about the role of antibiotics in URTI in children also showed similar results, with a total of 169 (34.9%) parents agreeing that most URTIs are viral in origin and self-limiting, requiring no antibiotic therapy.

Given that communities play a significant role in the emergence and spread of antibiotic resistance, the findings of this study somewhat agree with previous studies regarding antibiotic misuse. More than half of the participants in this survey (n = 394, 54.7%) still expect physicians to prescribe antibiotics when fever is present without considering the difference between viral and bacterial infections and how antibiotics are used in treating infections despite the fact that the majority of the participants know that antibiotics are used in bacterial infections. Furthermore, the degree of the adult Bisha population's awareness was made apparent with some statements associated with antibiotic misuse. Participants generally disagreed with the notion that antibiotics will make them recover more quickly when they have upper respiratory infections. Another interesting finding reported here is that participants know that the recurrent use of antibiotics leads to a decrease in their effectiveness and an increase in bacterial resistance, a finding that is the same as that reported by Shehadeh et al. [19] in Jordan in 2012.

Compared to other studies, in our study, 33.2% (n = 239) knew when it was appropriate to use antibiotics. For instance, a study conducted in Italy showed that 21.1% knew when to use antibiotics [20]. Also, nearly 81% were able to indicate that antibiotics are used to treat bacterial disease compared to a study conducted in Malaysia in which the percentage was 77% [21]. In the same study, it showed that 38% of the participants believed that the use of antibiotics would lead to quicker healing from typical cold symptoms [21], whereas our study showed a percentage of 64.1% (n = 462).

The population studied here generally agreed with statements of antibiotic misuse, which disagrees with some previously published studies. In Saudi Arabia, some studies have revealed that the population had good knowledge of antibiotic use. In contrast, a large-scale study investigated the knowledge, attitude, and practices (KAP) regarding antibiotics among Hajj pilgrims and reported that the pilgrims had some negative attitudes and poor practices, including acquiring and using antibiotics without a prescription, sharing antibiotics, using leftover antibiotics, and bringing antibiotics into Saudi Arabia from their country of origin [22-25].

Limitations

The cross-sectional study design may have limited the associations of the explored factors. Sociocultural and religious barriers affected the process of data collection in terms of the accuracy of data obtained and equal opportunity of participation for each individual in the community, which led to the use of a self-administered online questionnaire rather than an interviewer-administered questionnaire, which will allow us to reach a larger portion of the community (e.g., illiterate and people from lower socioeconomic status). Therefore, the possibility of bias limits the generalization of the results. In addition, children were not included, although they are more likely to develop URTI.

## Conclusions

Generally, there is poor knowledge of the appropriate use and the consequences of antibiotic abuse in upper respiratory tract infections and in the other areas assessed in the study, including causes and manifestations of URTIs. The results of this study revealed areas of misconceptions and specific groups to be targeted for educational interventions regarding the prudent use of antibiotics among the general adult population in the Bisha governorate. It is therefore suggested that a well-planned, organized, and structured educational program by public health professionals and policymakers, as well as medical school awareness campaigns organized by the University of Bisha medical students club, be undertaken to improve the awareness of the community regarding the correct indications and the harm of recurrent and irrational use of antibiotics.
